# Supplementary material for: Time-series transcriptome analysis identified differentially expressed genes in broiler chicken infected with mixed Eimeria species
Source: Front Genet. 2022 Aug 8;13:886781. doi: 10.3389/fgene.2022.886781 (PMC9393255; doi:10.3389/fgene.2022.886781)
Supplement: Supplementary file 2 [file DataSheet1.ZIP › 4dpi_KEGG.Gsea.1625100298584/KEGG_PORPHYRIN_AND_CHLOROPHYLL_METABOLISM.html]

Details for gene set KEGG\_PORPHYRIN\_AND\_CHLOROPHYLL\_METABOLISM[GSEA]

|  || Dataset | TMM\_4dpi\_gct\_format\_4dpi\_gct\_format.Class\_4dpi.cls #PC\_versus\_NC.Class\_4dpi.cls #PC\_versus\_NC\_repos |
| Phenotype | Class\_4dpi.cls#PC\_versus\_NC\_repos |
| Upregulated in class | 0 |
| GeneSet | KEGG\_PORPHYRIN\_AND\_CHLOROPHYLL\_METABOLISM |
| Enrichment Score (ES) | -0.59124595 |
| Normalized Enrichment Score (NES) | -1.7191932 |
| Nominal p-value | 0.013039568 |
| FDR q-value | 0.08632351 |
| FWER p-Value | 0.3586 |
Table: GSEA Results Summary

  

Fig 1: Enrichment plot: KEGG\_PORPHYRIN\_AND\_CHLOROPHYLL\_METABOLISM      
 Profile of the Running ES Score & Positions of GeneSet Members on the Rank Ordered List

  

| SYMBOL | TITLE | RANK IN GENE LIST | RANK METRIC SCORE | RUNNING ES | CORE ENRICHMENT || 1 | EARS2 | na | 1164 | 0.613 | -0.0195 | No |
| 2 | UROD | na | 4032 | 0.165 | -0.2377 | No |
| 3 | UROS | na | 4622 | 0.113 | -0.2725 | No |
| 4 | HCCS | na | 4707 | 0.106 | -0.2661 | No |
| 5 | HMBS | na | 4737 | 0.103 | -0.2555 | No |
| 6 | HMOX1 | na | 5931 | -0.002 | -0.3548 | No |
| 7 | GUSB | na | 6218 | -0.024 | -0.3756 | No |
| 8 | CPOX | na | 7636 | -0.147 | -0.4752 | No |
| 9 | COX15 | na | 8891 | -0.272 | -0.5453 | No |
| 10 | ALAD | na | 9443 | -0.341 | -0.5481 | Yes |
| 11 | ALAS1 | na | 9652 | -0.368 | -0.5188 | Yes |
| 12 | UGT2A1 | na | 10045 | -0.421 | -0.4982 | Yes |
| 13 | COX10 | na | 10242 | -0.451 | -0.4574 | Yes |
| 14 | CP | na | 10255 | -0.454 | -0.4010 | Yes |
| 15 | FTH1 | na | 11193 | -0.661 | -0.3955 | Yes |
| 16 | HMOX2 | na | 11235 | -0.676 | -0.3134 | Yes |
| 17 | BLVRA | na | 11540 | -0.831 | -0.2336 | Yes |
| 18 | FECH | na | 11552 | -0.841 | -0.1281 | Yes |
| 19 | UGT1A1 | na | 11907 | -1.313 | 0.0086 | Yes |
Table: GSEA details [plain text format]

  

Fig 2: KEGG\_PORPHYRIN\_AND\_CHLOROPHYLL\_METABOLISM      
 Blue-Pink O' Gram in the Space of the Analyzed GeneSet

  

Fig 3: KEGG\_PORPHYRIN\_AND\_CHLOROPHYLL\_METABOLISM: Random ES distribution      
 Gene set null distribution of ES for **KEGG\_PORPHYRIN\_AND\_CHLOROPHYLL\_METABOLISM**
